# Psychometric properties of Cognitive Instruments in Vascular Dementia and Alzheimer’s disease: a neuropsychological study

**DOI:** 10.6061/clinics/2020/e1435

**Published:** 2020-03-03

**Authors:** Juliana Francisca Cecato, Everton Balduino, Débora Fuentes, José Eduardo Martinelli

**Affiliations:** I Faculdade de Medicina de Jundiai, Jundiai, SP, BR; II Universidade Sao Francisco, Braganca Paulista, SP, BR

**Keywords:** Bender Gestalt Test, Dementia, Cognition, Neuropsychological Assessment, MMSE

## Abstract

**OBJECTIVES:**

To describe elderly performance in the Bender Gestalt Test (BGT) and to discriminate its score by using types of errors as comparison among healthy controls, Alzheimer’s disease (AD) patients, and vascular dementia (VD) patients.

**METHODS:**

We performed a cross-sectional analysis of 285 elderly individuals of both sexes, all over 60 years old and with more than 1 year of schooling. All participants were assessed through a detailed clinical history, laboratorial tests, neuroimaging, and neuropsychological tests including the BGT, the Cambridge Cognitive Examination (CAMCOG), the Mini-Mental State Examination (MMSE), the Geriatric Depression Scale (GDS), and the Pfeffer Functional Activities Questionnaire (PFAQ). The BGT scores were not used to establish diagnosis.

**RESULTS:**

Mean BGT scores were 3.2 for healthy controls, 7.21 for AD, and 8.04 for VD with statistically significant differences observed between groups (*p*<0.0001). Logistic regression analysis was used to identify the main risk factors for the diagnostic groups. BGT’s scores significantly differentiated the healthy elderly from those with AD (*p*<0.0001) and VD (*p*<0.0001), with a higher area under the curve, respectively 0.958 and 0.982. BGT’s scores also showed that the AD group presented 12 types of errors. Types of errors evidenced in the execution of this test may be fundamental in clinical practice because it can offer differential diagnoses between senescence and senility.

**CONCLUSION:**

A cut-off point of 4 in the BGT indicated cognitive impairment. BGT thus provides satisfactory and useful psychometric data to investigate elderly individuals.

## INTRODUCTION

The new diagnostic criteria for dementia strongly recommend the use of scales and neuropsychological instruments to evaluate dementia syndrome (1,2). The neuropsychological tests are important by virtue of their functional descriptions of cognitive operations (including memory, attention, executive function, and inhibitory control, among others) and were recommended as part of diagnostic investigations since cognitive impairment precedes brain damage (3,4). In contrast, the use of biomarkers in clinical practice is limited by high cost and obstacles faced as part of environmental and/or laboratory standardizations, as well as by the copyrights of ownership and/or methods of a number of laboratories, which increases costs and unavailability of biomarkers (2).

Cognitive models of drawing and copying, denominated as *praxis*, are widely used in psychological tests for cognitive assessments (5-7) and for personality traits (8). To mention just a few valid instruments that utilize such models for cognitive assessment, we can cite the Mini-Mental State Examination (MMSE), Cambridge Cognitive Examination (CAMCOG), and the Clock Drawing Test (CDT) (6,7,9-11). On the contrary, the absence of praxis (or apraxia), represents a wide spectrum of diseases characterized by an inability to perform an action or learned skill. There are several descriptions of apraxia, including loss of skill and manual dexterity, resulting in an inability to coordinate movements (12).

Accordingly, a specific test applied by psychologists is the Bender Gestalt Test (BGT) (13,14). Lauretta Bender designed the BGT in 1938 based on psychological laws of perceptual organization and stated that the reproduction/copy of drawings is not a simple learned task, but that it involves and depends on an appropriate neurological function for its implementation and success (15). Several studies have suggested the copy of drawings as a cognitive assessment instrument, allowing the diagnosis of dementia in the elderly (6,15-18). BGT is capable of assessing cognitive functions such as visuomotor ability, learning, memory and executive functions (19,20). In Brazil, the BGT was validated (21), followed by an update of the test that was published two years later (22).

An elegant study was able to differentiate Alzheimer’s disease (AD) and Lewy Body Dementia (LBD) through BGT scores, where the LBD group showed decreased BGT scores when compared with that of the AD group (18). The authors also found statistically significant differences when contrasting age ranges within the AD group. AD patients were divided into early (who started the dementia before age 65) and late (which developed after 65 years) dementia manifestation. These findings suggest different characteristics of cognitive impairment in these two groups *i.e.*, declines were observed in visual perceptual skills and executive functions in different ways between different age groups (18).

Therefore, the aim of this study was to describe psychometric properties of BGT in Alzheimer’s disease (AD) and vascular dementia (VD) in comparison to healthy controls (HC), and to determine which type of BGT execution errors frequently occur in cases of dementia. We also compared this instrument with MMSE and CAMCOG.

## METHODS

We conducted a cross-sectional study of 285 patients at the Geriatrics and Gerontology Department of the Jundiaí Medical School, from January 2015 to January 2018. The city of Jundiaí is located in the outskirts of the state of São Paulo, in the Southeastern region of Brazil. It has a population of approximately 414,810 inhabitants. It is the fifteenth most populous municipality in the state of São Paulo and it ranks seventh in the country in quality of life parameters for the elderly (23). In keeping with the criteria of our research, only patients diagnosed with Alzheimer’s disease (AD, n=170) or vascular dementia (VD, n=28), and healthy controls (HC, n=87) were selected, totaling a sample of 285 participants. The sample involved subjects from both genders, 60 years or older at the date of testing, and with at least one year of formal schooling.

HC participants fulfilled the following inclusion criteria: attended a follow-up medical consultation with geriatricians for maintenance of quality of life, presented no complaints of memory loss or behavioral changes, presented no diagnosis of psychiatric illness, and exhibited total independence in both basic and instrumental activities of daily life. AD participants had positive diagnosis for major neurocognitive disorder by Alzheimer's disease in accordance with National Institute on Aging - Alzheimer’s Association (2) and DSM-5 (24). Vascular dementia (VD) participants, on the other hand, were classified based on clinical criteria that conformed to Hachinski (25), NINDS-AIREN (26) and DSM-5 (24). The patients with a diagnosis of VD presented lacunar stroke, and neuroimaging was fundamental for differential diagnosis.

We excluded participants with severe dementia (CDR 3), depressive symptoms marked by the Geriatric Depression Scale’ score (GDS) higher than 5 points, upper limb tremors, paralysis, hearing impairment, severe visual impairment (which could compromise performance on cognitive instruments), and those that refused to complete any cognitive test. All procedures were carried out consonant with the research ethics committee of the institution and in accordance with the Declaration of Helsinki, receiving the opinion number identified as CEP 1.102.851, CAAE 42497414.3.0000.5412.

### Procedures

We collected behavioral, neurological and psychiatric data from clinical assessment and/or specific tests of each participant. Relatives and caregivers went through an interview in order to provide us with additional data regarding cognitive and functional features of the participants. Patients included in this study underwent complete laboratory exams (hemogram, vitamin B12, TSH, T4L, HIV and VDRL), which did not result in any pathological comorbidities. They also went through a MRI neuroimaging exam (gold standard test used mainly for VD diagnosis) in order to exclude other diagnostic possibilities (such as brain tumor or frontotemporal dementia) that justified the cognitive impairment. During a second meeting, they went through neurocognitive tests in a single session, lasting around 110 minutes. The diagnosis was determined after clinical, laboratory, neuroimaging, and neuropsychological analysis.

The neuropsychologist who administered the cognitive tests did not participate in the diagnostic process and was blind to any clinical information about the patients. The neuropsychological assessment consisted of the following instruments: CAMCOG, MMSE (which was part of the CAMCOG battery), GDS (Geriatric Depression Scale) (27) and Pfeffer Functional Activities Questionnaire (PFAQ) (28).

We did not use BGT scores to establish clinical diagnosis, but we used the PFAQ to assess daily live activities and GDS to detect depressive symptoms, combined with the MMSE (6) and CAMCOG (7) tests to evaluate cognition and to contribute to clinical diagnosis. CAMCOG has 67 items in its structure and evaluates memory, language, praxis, perception, abstract thinking, calculation, and orientation while BGT presents only praxis and perceptual organization evaluation.

The BGT results were analyzed through Lacks (20) criteria which considered 12 types of errors: rotation, overlapping difficulty, simplification, fragmentation, retrogression, perseveration, collision or collision tendency, impotence, closure difficulty, motor incoordination, angulation difficulty, and cohesion. The score ranged from 0 to 12 points, with a cut-off of ≤4 points, where lower scores indicate better cognitive performance. We added one point in the final score if the patient took more than 15 minutes to complete the test as indicated by Lacks’ criteria (20).

### Data Analysis

All data was analyzed by the IBM^®^ SPSS 20.0 (2011) software. We performed descriptive statistics to provide information on the sample distribution (age, sex, and years of schooling), followed by a sample distribution test using Kolmogorov-Smirnov and Shapiro-Wilk for cognitive tests (BGT, MMSE and CAMCOG) in order to verify if the sample corresponded to a parametric (age and CAMCOG) and non-parametric distribution (MMSE and BGT). Subsequently, we analyzed the statistical significance between the groups of schooling years and the scores of the cognitive tests establishing 5% as significance level. After that, we used Tukey’s tests for the variable age and Student’s t-test for the gender and schooling variables (Table 1). Categorical variables, as well as the evaluated scores of the instruments, were performed using chi-square (*x*^2^) and Kruskal-Wallis tests.

Correlation coefficients are commonly used for dementia diagnosis in Brazil to compare BGT’s scores with other instruments. We used ROC (Receiver Operating Characteristic) curve analysis to verify BGT’s sensitivity and specificity and to compare it with other diagnostic instruments (MMSE and CAMCOG). For that purpose, the software of choice was the MedCalc version 15.8 for Windows.

## RESULTS

The sample (Table 1) was composed of 285 participants classified according to the clinical diagnosis: 170 with AD (59.6%), VD (9.9%) and 87 HC (30.5%). For the healthy elderly group, the mean age was 75.31 years (min=60, max=93, SD=7.99), 75.9% (n=66) and 54% (n=47) from 1 to 4 years of schooling, 6.9% (n=6) from 5 to 8 years of schooling and 39.1% (n=34) ≥9 years of schooling. The group with AD presented a mean age of 78.03 years (min=60, max=100, SD=8.85), 62.9% (n=107) female sex and 67.7% n=115) between 1 and 4 years of schooling, 8.2% (n=14) between 5 and 8 years of schooling and 24.1% (n=41) ≥9 years of schooling. VD group presented a mean age of 75.32 years (min=62, max=91, SD=7.10), 57.1% (n=16) female and 82.1% (n=23) between 1 to 4 years of schooling, 3.6% (n=1) between 5 to 8 and more than 9 years 14.3% (n=4). There was no statistically significant difference in relation to age (*p*=0.202).

The research objective was to compare BGT to the instruments already validated and widely used in Brazilian elderly assessment. Therefore, we performed correlation analyses comparing the total sample and the AD group and observed a weak, but positive and significant coefficient correlation between the BGT score and age (r=0.20, *p*<0.0001) whereas a moderate, positive, and significant coefficient correlations were found between the BGT score and PFAQ (r=0.64, *p*<0.0001). We found a robust, negative, and significant coefficient correlation between MMSE (r=-0.72, *p*<0.0001) and CAMCOG (r=-0.75, *p*<0.0001). Negative correlations indicate inversely proportional quantities, which represent a satisfactory performance of the BGT. Elevated PFAQ scores indicate decline in living independence. Hence, we performed correlation analysis between the diagnostic groups and observed that the AD group presented a correlation between the BGT scores and age (r=0.13, *p*=0.098). There was a moderate, positive and significant correlation when compared to PFAQ (r=0.32, *p*<0.0001), and a moderate, negative and significant correlation was observed between MMSE (r=-0.54, *p*<0.0001) and CAMCOG (r=-0.60, *p*<0.0001).

In VD groups, there was no significant correlation between the BGT score and age (r=0.10; *p*=0.594). We found significant, moderate, positive correlation between PFAQ (r=0.46; *p*=0.010), and significant, moderate, and negative correlations between MMSE (r=-0.45; *p*=0.016), CAMCOG (r=-0.48; *p*=0.010) and CDT (r=-0.53; *p* =0.005).

**Figure 1:**
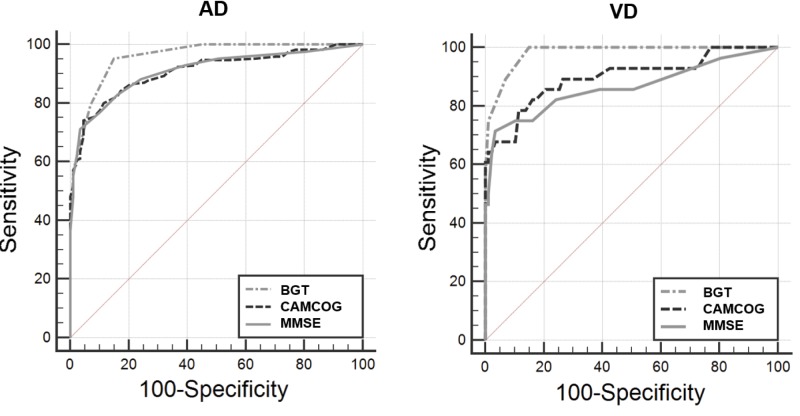
Graphical analysis of the ROC curve between control and elderly subjects with AD groups showing comparisons between cognitive instruments. AD group: representation of the largest area under the curve (AUC) evidenced by the Bender Gestalt Test, followed by the MMSE (B) and CAMCOG (C). VD: representation of the largest area under the curve (AUC) evidenced by the Bender Gestalt Test, followed by CAMCOG and MMSE.

Table 2 represents the sensitivity and specificity data of the instruments. It was verified that the instrument with the largest area under the curve (AUC) was the BGT in the AD group (AUC=0.958) with sensitivity and specificity of 95% and 85%, respectively, with a cut-off point of 4 in the BGT, which identified cognitive impairment. The BGT instrument presented AUC=0.982 in the VD group. Table 2 and Figure 1 demonstrate the performance of the cognitive instruments in both groups. BGT was the instrument with the highest AUC, indicating satisfactory psychometric properties to identify cognitive deficits compatible with dementia. The MMSE was the instrument that presented the second most satisfactory result (AUC=0.910), with sensitivity of 71% and specificity of 96%, for a cut-off score of 23 points. CAMCOG exhibited the third highest AUC (0.908), with sensitivity of 74% and specificity of 95%, for a cut-off point of 76 points. These results suggest that for the diagnostic investigation of AD, BGT has the greatest capacity to identify cognitive deficit and exclude healthy patients (Table 2 and Figure 1). We observed similar results in the VD group.

Table 3 indicates that AD and VD patients present a significantly higher number of errors in the execution of BGT when compared to normal controls. To analyze these results, we performed Pearson's Chi-square test and compared the percentages of the groups, obtaining the statistical significance. Our analysis indicated that virtually all type of errors would result in important differences in the BGT scores between VD, AD, and HC.

## DISCUSSION

BGT is one of the most widely used instruments by professional psychologists in clinical environments, mainly used in the evaluation of children. In terms of praxis, the condition of impairment in motor activities that are not related to muscle weakness (apraxia) may represent neurological injury or dementia. There might be praxis impairment in dementia and it may be confused with apathy or depression, since many family members notice alterations in the patient’s behavior and report them as lack of interest in previously preferred activities. The incapability to perform an action due the apraxia condition might be the cause for this loss of interest. Praxis status might be assessed through tests such as the MMSE (with the pentagon design) and/or the CAMCOG (with the copy of drawings and the design of a clock). Rey's Complex Figure (RCF) is widely used with satisfactory results in diagnostic investigations of the patients’ praxis with suspected cerebral dysfunction (29). The limitation of RCF is that the instrument does not identify the types of errors committed by the patient, while the BGT is capable of such identifications. In this study, we sought to compare the performance of the BGT, in consonance with the analysis of the types of errors as proposed by Lacks’s criteria (20), adapted from a Huttin-Briskin scale, in healthy elderly individuals and in patients with AD.

To our knowledge, this is the first study to explore the psychometric contribution of BGT with a version of the scoring system based on 12 errors in patients with AD and VD. A similar study sought to investigate the performance of BGT in elderly subjects with LBD and AD (18). However, the aforementioned study had a sample of 36 patients with AD and 18 with LBD, while our sample included 257 elderly subjects, of which 170 had an AD diagnosis.

Therefore, it became evident that patients with AD or VD committed several errors in their BGT execution (Table 3). Praxis is one of the main cognitive functions that can predict dementia diagnosis (30,31). Both praxis and memory have shown to be determinant in the assessment of patients with AD due to cortical degeneration related to memory and praxis impairment.

Previous evaluation of AD patients reported errors in intersection, closure, rotation, and closing-in of the MMSE drawings (32). It was also reported that perseveration and simplification errors occur in patients with dementia using the Weschler Adult Intelligence Scale (33). These studies corroborate our findings, where we considered that the type of errors made by patients with AD may be an indication of cortical dementia. There are indications of cortical lesions in AD characterized by dementia (30,32) and therefore, the instruments that evaluate praxis, especially the ones that measure the type of errors, can indicate an evolving dementia. The assessment of the type of errors is the main advantage of BGT, since the same test can indicate the errors mentioned in these studies (32,33), even though the authors of both studies used two different instruments to obtain the same outcome. BGT presents in its structure the errors cited by several authors, which can offer great help in differential diagnoses.

The importance of the type of error analysis in praxis assessment is justified by the confirmation that cognitive function impairments in AD show alterations in the “regional cerebral blood flow” (rCBF) exam (30). The study showed that both the upper and lower right parietal lobes were associated with the highest damage in constructive praxis, evidenced by correlation analysis, respectively, r=0.613 and r=0.699. Similar to the parietal lobe, the results pointed out that the right upper temporal lobe was correlated with constructive praxis and orientation. It was also shown that constructive praxis correlated with the angular gyrus (r=0.618) and cingulate gyrus regions bilaterally (Right r=0.573; Left r=0.557). Interestingly, there is a significant relationship between the decline in visuoconstruction and the losses in the right upper parietal lobe, right lower parietal lobe, right medial temporal lobe, and cingulate gyrus (30). Other cognitive functions (such as memory and language) showed impairment in fewer brain regions such as the left medial temporal lobe and angular gyrus, while memory, language and constructive praxis correlated with their losses or damages.

Our findings are consistent with the praxis decline in AD and VD as demonstrated by another study (30). We presented sensitivity and specificity data from BGT and compared it with other instruments, such as MMSE and CAMCOG, showing that the BGT has the highest AUC for AD and VD comparisons (AUC=0.958 and 0.982, respectively). Another key point of the aforementioned study was the use of a cognitive test battery to assess several cognitive functions, demonstrating that the constructive praxis was most related to the decrease in cerebral blood flow examined by the rCBF exam (30). These findings contribute to evidence supporting our study’s results where we sought to describe the BGT's psychometric properties with scores for analysis that considers 12 types of errors (20), aiming to assess constructive praxis impairments in patients with AD and VD. In our analysis, the BGT presented the highest AUC when compared to CAMCOG and MMSE primarily because constructive praxis depends on several brain areas for its proper execution and its impairment may suggest neurofunctional and neurostructural damage, as expected to occur in AD and VD (30).

Our study was limited by the number of patients in the VD group, due to the exclusion criteria and the fact that many patients who were affected by ischemic injury exhibited severe motor impairments, making it impossible to perform visuoconstructive praxis tasks.

## CONCLUSION

Schooling years are a determinant factor for neuroprotection against dementia syndrome. Whenever apraxia symptoms occur in neuropsychological evaluations, it may indicate the presence of a neurodegenerative disease. Here, we present accuracy data from an important instrument for praxis evaluation in elderly subjects.

Visuoconstructive skills have been shown to be correlated with cases of faster dementia progression as described by Hazan et al. (34). This can clarify the aggressive progression of dementia in patients that showed early praxis decline, since this association may be explained by degeneration of temporal and parietal areas, i.e. brain areas that are involved in the circuitry underlying praxis. This hypothesis could explain the slower progression of dementia in some patients even while others exhibit faster progression (35).

In this study, the instrument BGT presented the best diagnostic accuracy in distinguishing healthy elderly subjects from those diagnosed with AD and VD. The Lacks’ criteria (20), suggesting a consideration of the type of errors to be satisfactory in differentiating the groups (*p*<0.0001), together with our findings, may contribute to the analysis of the praxis dysfunction caused by the neurological alterations in the dementia syndrome. With these findings, we suggest using the BGT for assessing cognitive deficits in AD and VD patients.

## AUTHOR CONTRIBUTIONS

Cecato JF designed the study, collected the data, participated in statistical analysis and wrote the manuscript. Balduino E participated in statistical analysis. Fuentes D participated in data collection. Martinelli JE revised the manuscript.

## Figures and Tables

**Table 1:** Characteristics of the sample according to age, sex and schooling.

	Controls	AD	VD	*p*
Years old	75.31±7.99	78.03±8.85	75.32±7.10	*0.202
**Education (number of subjects)**				
1 to 4 years	47	115	23	
5 to 8 years	6	14	1	**0.0001
>9 years	34	41	4	
CAMCOG	89.8±8.5	65.7±15.3	62.07±19.73	
MMSE	27.7±2.1	20.8±4.7	20.32±6.08	**0.0001
BGT	3.2±1.4	7.21±2.01	8.04±6.08	

**Table 2:** Sensitivity and specificity data according to the ROC curve methodology, in AD and VD groups. Sen.=sensitivity; Spe.=Specificity; AUC=area under the curve; CI=confidence interval. *p*=*x* ^2^.

Instruments	AUC	*p*	Sen. (%)	IC (95%)	Spe. (%)	CI (95%)	Cut-off points
**AD group**							
BGT	0.958	<0.0001	95	90.9 - 97.9	85	75.8 - 91.8	4 points
MMSE	0.910	<0.0001	71	63.7 - 77.9	96	90.3 - 99.3	23 points
CAMCOG	0.908	<0.0001	74	66.9 - 80.5	95	88.6 - 98.7	76 points
**VD group**							
BGT	0.982	<0.0001	100	87.7 - 100	85	75.8 - 91.8	4 points
CAMCOG	0.898	<0.0001	78	59.0 - 91.7	88	79.9 - 94.3	79 points
MMSE	0.865	<0.0001	71	51.3 - 86.8	96	90.3 - 99.3	23 points

**Table 3:** Frequencies, percentages and p-values for types of errors.

Types of erros	HC (N=87)	AD (N=170)	VD (N=28)	*p*
Rotation	5 (5.7%)	54 (31.8%)	15 (53.6%)	0.0001
Overlapping difficulty	49 (56.3%)	140 (82.4%)	25 (89.3%)	0.0001
Simplification	37 (42.5%)	145 (85.3%)	27 (96.4%)	0.0001
Fragmentation	5 (5.7%)	105 (61.8%)	16 (57.1%)	0.0001
Retrogression	9 (10.3%)	87 (51.2%)	19 (67.9%)	0.0001
Perseveration	47 (54.0%)	143 (84.1%)	25 (89.3%)	0.0001
Collision or collision tendency	24 (27.6%)	81 (47.6%)	11 (39.3%)	0.0020
Impotence	8 (9.2%)	45 (26.5%)	7 (25.0%)	0.0012
Closure difficulty	37 (42.5%)	153 (90.0%)	25 (89.3%)	0.0001
Motor incoordination	19 (21.8%)	83 (48.8%)	16 (57.1%)	0.0001
Angulation difficulty	43 (49.4%)	141 (82.9%)	26 (92.9%)	0.0001
Cohesion	5 (5.7%)	39 (22.9%)	10 (35.7%)	0.0005
